# 

**Published:** 1894-03

**Authors:** 


### CONTENTS.

ORIGINAL ARTICLES—

Sulphur in the Treatment of Pityriasis Capitis. By Bernard Wolff, M. D., Atlanta, Ga. .......... 117
Acute Pneumonia in Childhood. By Thomas S. Southworth, M. D., New York .......... 122

SOCIETY REPORTS—

Clinical Society of Maryland .......... 136

BOOK REVIEWS—

American Text-book of Gynecology .......... 142
Stricture of the Urethra. By G. Frank Lydston, M. D., Chicago .......... 142

SELECTIONS AND ABSTRACTS—

Sir Andrew Clark—A Reminiscence. By Frances E. Willard .......... 143
Laminectomy for Spinal Injuries .......... 145
The Treatment of Burns. By J. W. Lindsey, Claysburg, Pa. .......... 147

SELECTIONS AND ABSTRACTS—CONTINUED—

Proper Method of Percussing the Chest. By Thomas J. Mays, A. M., M. D., Philadelphia ........ 149
Subacute Unilateral Bulbar Palsy ........ 151
A New Operation for Vesico-Vaginal Fistula ........ 152

EDITORIAL—

Smallpox, with Its Prevention by General Vaccination ........ 153

EDITORIAL NOTES—

Thirty-sixth Annual Commencement of the Atlanta Medical College. 159
Letter upon Yellow Fever Inoculation ........ 161

PRESCRIPTION DEPARTMENT ........ 166, 167

Epistaxis—Hiccough—A Cathartic Lemonade—Ovarian Neuralgia—Rheumatic Fever—Congestive and Inflammatory Conditions of the Ear—Purgative Combination for Children—For Irritable Bladder—Epilepsy—LaGrippe.

SPECIAL NOTES ........ 168–174

